# Psoriasis treat to target: defining outcomes in psoriasis using data from a real‐world, population‐based cohort study (the British Association of Dermatologists Biologics and Immunomodulators Register, BADBIR)

**DOI:** 10.1111/bjd.18333

**Published:** 2019-09-10

**Authors:** S.K. Mahil, N. Wilson, N. Dand, N.J. Reynolds, C.E.M. Griffiths, R. Emsley, A. Marsden, I. Evans, R.B. Warren, D. Stocken, J.N. Barker, A.D. Burden, C.H. Smith, the BADBIR study group and the PSORT consortium

**Affiliations:** ^1^ St John's Institute of Dermatology Guy's and St Thomas’ NHS Foundation Trust London U.K.; ^2^ Institute of Health and Society Faculty of Medical Sciences Newcastle University Newcastle upon Tyne U.K.; ^3^ Department of Medical and Molecular Genetics King's College London London U.K.; ^4^ Dermatological Sciences Institute of Cellular Medicine Medical School Newcastle University Newcastle upon Tyne U.K.; ^5^ Department of Dermatology Royal Victoria Infirmary Newcastle Hospitals NHS Foundation Trust Newcastle upon Tyne U.K.; ^6^ Dermatology Centre Salford Royal NHS Foundation Trust University of Manchester Manchester Academic Health Science Centre NIHR Manchester Biomedical Research Centre Manchester U.K.; ^7^ Department of Biostatistics & Health Informatics Institute of Psychiatry, Psychology and Neuroscience, King's College London London U.K.; ^8^ Centre for Biostatistics School of Health Sciences University of Manchester Manchester Academic Health Science Centre Manchester U.K.; ^9^ Clinical Trials Research Unit Leeds Institute of Clinical Trials Research University of Leeds Leeds U.K.; ^10^ Institute of Infection, Immunity and Inflammation University of Glasgow Glasgow U.K.

## Abstract

**Background:**

The ‘treat to target’ paradigm improves outcomes and reduces costs in chronic disease management but is not yet established in psoriasis.

**Objectives:**

To identify treatment targets in psoriasis using two common measures of disease activity: Psoriasis Area and Severity Index (PASI) and Physician's Global Assessment (PGA).

**Methods:**

Data from a multicentre longitudinal U.K. cohort of patients with psoriasis receiving systemic or biologic therapies (British Association of Dermatologists Biologics and Immunomodulators Register, BADBIR) were used to identify absolute PASI thresholds for 90% (PASI 90) and 75% (PASI 75) improvements in baseline disease activity, using receiver operating characteristic curves. The relationship between PGA (clear, almost clear, mild, moderate, moderate–severe, severe) and PASI (range 0–72) was described, and the concordance between absolute and relative definitions of response was determined. The same approach was used to establish treatment response and eligibility definitions based on PGA.

**Results:**

Data from 13 422 patients were available (58% male, 91% white ethnicity, mean age 44·9 years), including over 23 000 longitudinal PASI and PGA scores. An absolute PASI ≤ 2 was concordant with PASI 90 and an absolute PASI ≤ 4 was concordant with PASI 75 in 90% and 88% of cases, respectively. These findings were robust to subgroups of timing of assessment, baseline disease severity and treatment modality. PASI and PGA were strongly correlated (Spearman's rank correlation coefficient 0·92). The median PASI increased from 0 (interquartile range 0–0, range 0–23) to 19 (interquartile range 15–25, range 0–64) for PGA clear to severe, respectively. PGA clear/almost clear was concordant with PASI ≤ 2 in 90% of cases, and PGA moderate–severe severe was concordant with the National Institute for Health and Care Excellence PASI eligibility criteria for biologics in 81% of cases.

**Conclusions:**

An absolute PASI ≤ 2 and PGA clear/almost clear represent relevant disease end points to inform treat‐to‐target management strategies in psoriasis.

**What's already known about this topic?**

- The most commonly used relative disease activity measure in psoriasis is ≥ 90% improvement in Psoriasis Area and Severity Index (PASI 90); however, it has several limitations including dependency on a baseline severity assessment.
- Defining an absolute target disease activity end point in psoriasis has the potential to improve patient outcomes and reduce costs, as demonstrated by treat‐to‐target approaches in other chronic diseases such as hypertension and diabetes.
- The Physician's Global Assessment (PGA) is a popular alternative measure of psoriasis severity in daily practice; however, its utility has not been formally assessed with respect to PASI.

**What does this study add?**

- An absolute PASI ≤ 2 corresponds with PASI 90 response and is a relevant disease end point for treat‐to‐target approaches in psoriasis.
- There is a strong correlation between PASI and PGA.
- PGA moderate–severe/severe may serve as an alternative eligibility criterion for biologics to PASI‐based definitions, and PGA clear/almost clear is an appropriate alternative absolute treatment end point.

**What are the clinical implications of this work?**

- Absolute PASI ≤ 2 and PGA clear/almost clear represent relevant disease end points to inform treat‐to‐target management strategies in psoriasis.

## Abstract 2

**Linked Editorial:** Takeshita. *Br J Dermatol* 2020; 182:1075–1076.

Psoriasis is a chronic, inflammatory skin disease that is recognized as a major global health problem by the World Health Organization and affects 2–4% of the population.^1^ It is associated with reduced quality of life and multiple morbidities including psoriatic arthritis, cardiovascular disease, obesity and depression.^2^ Recent insights into the molecular pathogenesis of psoriasis have led to the development of increasingly effective targeted therapies, which have transformed patient and clinician expectations of treatment^3^ and improved comorbidity outcomes.^4^ In this context, a robust target disease activity end point is needed to drive the introduction and modification of treatments in a timely, effective and cost‐efficient manner. This ‘treat to target’ paradigm is well established in cardiology (hypertension, hyperlipidaemia), endocrinology (diabetes mellitus) and rheumatology (rheumatoid arthritis), and can improve patient outcomes and reduce costs.^5^, ^6^

In psoriasis, the disease activity end point is currently defined by a relative change from baseline rather than an absolute measure, using the Psoriasis Area and Severity Index (PASI; range 0–72).^6^, ^7^, ^8^ PASI 75 (≥ 75% improvement in PASI from baseline) and, increasingly, PASI 90, are common primary end points in interventional clinical trials^7^ and parallel clinically relevant improvements in patient‐reported outcomes [Dermatology Life Quality Index (DLQI) 0 or 1].^8^ These end points have driven treatment guideline recommendations on eligibility and response to psoriasis interventions.^9^, ^10^ However, in clinical practice the accuracy and relevance of relative (in contrast to absolute) PASI measures are limited by a dependence on the baseline PASI. This may have been established historically or be uncertain due to the chronic nature of the disease and variability in washout of prior systemic therapies. There is also potential for interassessor variability of current vs. baseline PASI measurements.

Aside from difficulties related to use of a baseline assessment to inform a treatment target, there are also limitations with the measure itself. Separate calculations of extent and intensity of plaques of psoriasis at four anatomical regions (head, trunk, and upper and lower extremities) may be challenging in time‐pressed routine practice^11^ and introduce potential for interassessor variation and calculation errors. There is limited sensitivity for evaluating patients with low levels of disease, redundancy at the upper half of the range for PASI and a paucity of data on the utility of PASI in real‐world as opposed to trial settings.^12^ The Physician's Global Assessment (PGA) may be a useful alternative measure for daily practice, as it is a simple, average assessment of all psoriasis lesions according to a Likert scale (six‐point score in the European Medicines Agency guidelines: clear, almost clear, mild, moderate, moderate–severe, severe).^13^ However, there has been no formal assessment of its utility with respect to PASI in routine practice.

In this study we used a large‐scale real‐world multicentre longitudinal cohort of patients receiving systemic therapies (the British Association of Dermatologists Biologics and Immunomodulators Register, BADBIR)^14^ to establish an absolute definition of disease control based on the most widely used relative measure, PASI 90. The clinical utility of PGA was investigated by determining the relationship between PASI and PGA, and exploring how this relates to current PASI‐based definitions of treatment eligibility and response using the U.K. exemplar National Institute for Health and Care Excellence (NICE) criteria.^9^

## Patients and methods

### Study design and setting

This study uses data from BADBIR, which is a U.K. and Republic of Ireland multicentre pharmacovigilance registry (research ethics committee reference 07/MRE08/9). BADBIR was established in 2007 for individuals with psoriasis starting on systemic therapies and aged 16 years or older, and is described in detail elsewhere (http://www.badbir.org).^14^, ^15^ It includes detailed demographic and longitudinal clinical data on all participants including regular multimodal disease severity and treatment outcome measurements such as PASI and PGA. Baseline assessments are completed within the first 6 months of treatment (−183 to 0 days) and follow‐up visits are at 6‐monthly intervals for the first 3 years and then annually to 10 years. The data cutoff for this analysis is 1 April 2018.

Individuals with a diagnosis of psoriasis under the care of a dermatologist, started on or switched to a biologic (for the ‘biologic cohort’) or nonbiologic systemic therapy (for the ‘nonbiologic systemic cohort’) within the previous 6 months and able to give informed consent are eligible for inclusion. Individuals in the ‘nonbiologic systemic cohort’ have PASI ≥ 10 and DLQI >10 (unless switching between nonbiologic systemic agents) and have no prior exposure to a biologic agent.^14^ There are no minimum PASI or DLQI inclusion criteria for participants in the ‘biologic cohort’, as eligibility for biologic therapy according to the NICE criteria is assumed (PASI ≥ 10 and DLQI > 10).^9^

### Outcome measures

PASI scores are measured consecutively over time for each participant, and the primary outcome measure of treatment response is defined as PASI 90, which is the most widely used standard primary outcome in psoriasis. PASI 75 response is analysed as a secondary outcome.

### Statistical methods

#### Identifying an absolute Psoriasis Area and Severity Index (PASI) threshold that corresponds to PASI 90 and PASI 75

Receiver operating characteristic (ROC) curves are used on the longitudinal PASI data in BADBIR to establish absolute PASI thresholds corresponding to PASI 90 and PASI 75 responses. Individuals with a missing baseline PASI are not included. As PASI 90 and PASI 75 are calculated with respect to baseline PASI, mathematical coupling^16^ between the relative and absolute PASI values would inflate the statistical measures derived from the ROC curve. Therefore, ROC curves are used to inform cut point locations based on maximizing the sum of sensitivity and specificity with bootstrap estimation (100 bootstrap replications). Contingency tables then identify clinically relevant cut points within the identified locations. Cohen's kappa is used to quantify agreement with PASI‐based definitions. This statistic is inflated due to the definitions of response being based on the same PASI value, so should be treated with caution. Cohen's kappa 0·41–0·60, 0·61–0·80 and > 0·80 indicate moderate, substantial and almost perfect agreements, respectively.

Sensitivity analyses were used to investigate the impact of treatment (biologic vs. nonbiologic systemic agents), timing of assessment (6 months vs. 12 months following start of treatment; 2013–2015 vs. 2016–2018; baseline PASI assessment on treatment start date vs. prior to treatment start date) and baseline disease severity (PASI < 10, 10–20, > 20). The time periods 2013–2015 and 2016–2018 were selected to assess for any effect of the recent introduction of more efficacious biologic agents.

#### Exploring the relationship between Psoriasis Area and Severity Index and Physician's Global Assessment (PGA), and establishing PGA response and eligibility definitions

The relationship between PASI and PGA was assessed graphically using box and whisker plots and quantified using Spearman's rank correlation coefficient. The approach described above was used to evaluate the potential impact of changing from PASI to PGA in clinical practice. We thus used the absolute PASI threshold corresponding to the most commonly used relative PASI definition of treatment response (PASI 90)^13^ and PASI‐based NICE criteria for biologics eligibility (PASI ≥ 10)^9^ to derive PGA‐based definitions for response and biologic eligibility. Cohen's kappa was used to quantify agreement between PASI‐ and PGA‐based definitions. All patients included in the analysis had both a PGA and PASI recorded on the same day postbaseline. All analyses are on a complete‐case basis and were conducted in Stata 15 (StataCorp, College Station, TX, U.S.A.).^17^

## Results

### Cohort characteristics

Data from 13 422 patients with psoriasis enrolled in BADBIR are included. Of these, 9201 patients received a biologic agent (‘biologic cohort’) and 4221 patients received a nonbiologic systemic treatment (‘nonbiologic systemic cohort’) (Table 1; and Table S1; see Supporting Information). In total 1371 patients switched from the nonbiologic systemic cohort to the biologic cohort and are therefore included in the analysis twice. Hence, data from 12 051 patients are unique. Patients had consecutive outcome assessments performed over a median of 338 days (interquartile range 252–386, range 0–7530).

**Table 1:** Baseline characteristics of the BADBIR cohort

	Biologic cohort (*n* = 9201)	Nonbiologic systemic cohort (*n* = 4221)	Overall (*n* = 13 422)^a^
Disease duration (years), mean ± SD	21·7 ± 12·6	18·7 ± 13·3	20·7 ± 12·9
	*n* = 9134	*n* = 4202	*n* = 13 336
Age of onset (years), mean ± SD	23·3 ± 13·5	25·5 ± 15·3	24·0 ± 14·1
	*n* = 9185	*n* = 4216	*n* = 13 401
Baseline PASI score, mean ± SD	15·7 ± 8·1	14·8 ± 8·0	15·4 ± 8·1
	*n* = 7948	*n* = 3957	*n* = 11 905
Baseline DLQI score, mean ± SD	17·4 ± 7·7	15·6 ± 7·1	16·7 ± 7·5
	*n* = 4683	*n* = 3054	*n* = 7737
Baseline PGA score	*n* = 6584	*n* = 3593	*n* = 10 177
Severe	2111 (32·1)	870 (24·2)	2981 (29·3)
Moderate–severe	2869 (43·6)	1510 (42·0)	4379 (43·0)
Moderate	1312 (19·9)	924 (25·7)	2236 (22·0)
Mild	180 (2·7)	198 (5·5)	378 (3·7)
Almost clear	81 (1·2)	71 (2·0)	152 (1·5)
Clear	31 (0·5)	20 (0·6)	51 (0·5)
Sex male	5455 (59·3)	2366 (56·1)	7821 (58·3)
	*n* = 9201	*n* = 4221	*n* = 13 422
White ethnicity	8391 (91·4)	3800 (90·4)	12 191 (91·1)
	*n* = 9179	*n* = 4202	*n* = 13 381
Age (years), mean ± SD	45·1 ± 13·1	44·3 ± 14·4	44·9 ± 13·5
	*n* = 9201	*n* = 4221	*n* = 13 422
Body mass index (kg m^−2^)	*n =* 8585	*n =* 3912	*n =* 12 497
Mean ± SD	31·1 ± 7·2	30·2 ± 7·1	30·8 ± 7·2
Underweight (< 18·5)	71 (0·8)	56 (1·4)	127 (1·0)
Normal weight (18·5–24·9)	1530 (17·8)	864 (22·1)	2394 (19·2)
Overweight (25·0–29·9)	2718 (31·7)	1296 (33·1)	4014 (32·1)
Obese class I (30·0–34·9)	2154 (25·1)	900 (23·0)	3054 (24·4)
Obese class II (35·0–39·9)	1199 (14·0)	423 (10·8)	1622 (13·0)
Obese class III (≥ 40·0)	913 (10·6)	373 (9·5)	1286 (10·3)
Smoking status	*n* = 8019	*n* = 3711	*n* = 11 730
Never smoked	2724 (34·0)	1124 (30·3)	3848 (32·8)
Previously smoked	2902 (36·2)	1328 (35·8)	4230 (36·1)
Currently smokes	2393 (29·8)	1259 (33·9)	3652 (31·1)
Chronic plaque psoriasis	9098 (98·9)	4169 (98·8)	13 267 (98·8)
	*n* = 9201	*n* = 4221	*n* = 13 422
Psoriatic arthritis at baseline	2096 (22·8)	441 (10·4)	2537 (18·9)
	*n* = 9201	*n* = 4221	*n* = 13 422
Other type(s) of psoriasis^b^	2210 (24·1)	1105 (26·2)	3315 (24·8)
	*n* = 9169	*n* = 4214	*n* = 13 383

The data are presented as *n* (%) unless stated otherwise. PASI, Psoriasis Area and Severity Index; DLQI, Dermatology Life Quality Index; PGA, Physician's Global Assessment. ^a^1371 patients switched from a nonbiologic systemic to a biologic agent so are included in these summaries twice. There are therefore 12 051 unique patients overall. ^b^Erythrodermic, guttate, generalized pustular, localized pustular or unstable.

The baseline characteristics of the participants are listed in Table 1 and are in line with those from previous reports.^15^, ^18^ The average baseline PASI was 15·4 ± 8·1 and 58·3% are male. The average age and body mass index of participants are 44·9 ± 13·5 years and 30·8 ± 7·2 kg m^−2^, respectively. Almost all participants have chronic plaque psoriasis (98·8%) and 18·9% have concurrent psoriatic arthritis.

### Psoriasis Area and Severity Index (PASI) ≤ 2 is consistent with PASI 90 response

Response status (i.e. responder or nonresponder) was assigned according to PASI 90 status. The relative PASI response was derived from 23 501 longitudinal PASI measurements in 10 894 patients, in which each patient has a baseline PASI and at least one follow‐up PASI recorded. When balancing sensitivity and specificity, the ROC curve analysis indicates that an absolute PASI threshold around 1·6 [95% confidence interval (CI) 1·5–1·7] is consistent with PASI 90 response. To optimize potential practical utility, we explored absolute PASI thresholds of 2 and 1·5 (Table 2).

**Table 2:** Comparison of absolute Psoriasis Area and Severity Index (PASI) definitions of response with PASI 90 and PASI 75. These data are derived from 23 501 PASI measurements in 10 894 patients

		PASI 90	
		No	Yes
PASI ≤ 1·5	No	14 817 (63)	606 (3)
	Yes	979 (4)	7099 (30)
PASI ≤ 1·6	No	14 622 (62)	506 (2)
	Yes	1174 (5)	7199 (31)
PASI ≤ 2^a^	No	13 619 (58)	236 (1)
	Yes	2177 (9)	7469 (32)
		PASI 75	
		No	Yes
PASI ≤ 3	No	9814 (42)	1386 (6)
	Yes	1105 (5)	11 196 (48)
PASI ≤ 3·3	No	9532 (41)	1092 (5)
	Yes	1387 (6)	11 490 (49)
PASI ≤ 3·5	No	9359 (40)	951 (4)
	Yes	1560 (7)	11 631 (49)
PASI ≤ 4^b^	No	8737 (37)	582 (2)
	Yes	2182 (9)	12 000 (51)

The data are presented as *n* (%). ^a^For PASI 90 and PASI ≤ 2: agreement 90%, Cohen's kappa 0·78 (95% confidence interval 0·77–0·79). ^b^For PASI 75 and PASI ≤ 4: agreement 88%, Cohen's kappa 0·76 (95% confidence interval 0·75–0·77).

**Figure 1:**
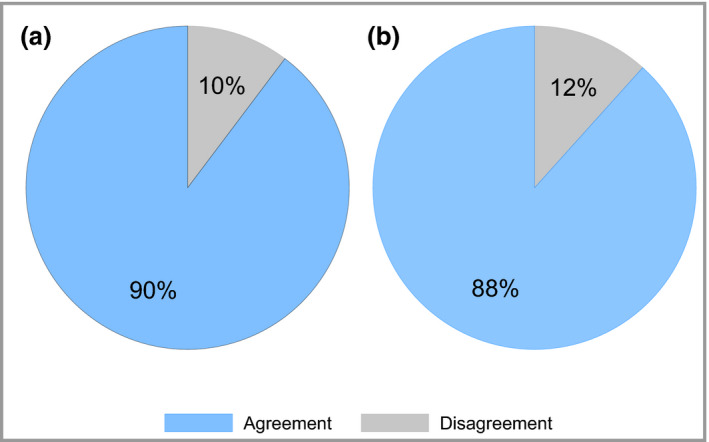
Agreement between (a) ≥ 90% improvement in Psoriasis Area and Severity Index (PASI 90) and absolute PASI ≤ 2, and (b) PASI 75 and PASI ≤ 4. The blue segment represents the agreement between the two definitions and the grey segment represents the disagreement. These data are derived from 23 501 longitudinal PASI measurements in 10 894 patients.

PASI ≤ 2 assigns the same response status as PASI 90 in 90% of cases, with a Cohen's kappa of 0·78 (95% CI 0·77–0·79), indicating substantial agreement (Table 2, Fig. 1a). PASI ≤ 1·5 assigns the same response status as PASI 90 in 93% of cases (Cohen's kappa 0·85, 95% CI 0·84–0·86); however, 3% of cases are classified as nonresponders using PASI ≤ 1·5 but are responders according to PASI 90 status. This reduces to 1% using PASI ≤ 2, indicating a more frequent correct assignment of PASI 90 response status (i.e. fewer false negative classifications) with this higher absolute threshold. These PASI 90 responders who are classified as nonresponders according to an absolute PASI threshold of 2 necessarily have more severe disease (mean ± SD baseline PASI 33.5 ± 8.9 vs. mean baseline PASI 15.4 ± 8·1 for the whole cohort).

Overall, 4% of cases are classified as responders using PASI ≤ 1·5 but are nonresponders according to PASI 90. This increases to 9% for PASI ≤ 2 as this higher threshold is more liberal for assigning responder status. Cases classified in this way using PASI ≤ 2 necessarily have milder disease pretreatment (mean baseline PASI 10.2 ± 4·2 vs. mean baseline PASI 15.4 ± 8·1 for the whole cohort). This highlights the inherent mathematical constraints of achieving a relative PASI 90 response with a low baseline PASI.

### Psoriasis Area and Severity Index ≤ 4 is consistent with PASI 75 response

The ROC curve analysis indicates that an absolute PASI threshold around 3·3 (95% CI 3·0–3·5) is concordant with PASI 75 response. We therefore explored the more practical absolute definitions of PASI ≤ 3, PASI ≤ 3·5 and PASI ≤ 4 (Table 2). By applying the same logic as described above for PASI 90, an absolute PASI ≤ 4 was identified as concordant with PASI 75. The agreement between PASI 75 and PASI ≤ 4 is 88%, with a Cohen's kappa 0·76 (95% CI 0·75–0·77) (Fig. 1b).

### Psoriasis Area and Severity Index correlates with Physician's Global Assessment

**Figure 2:**
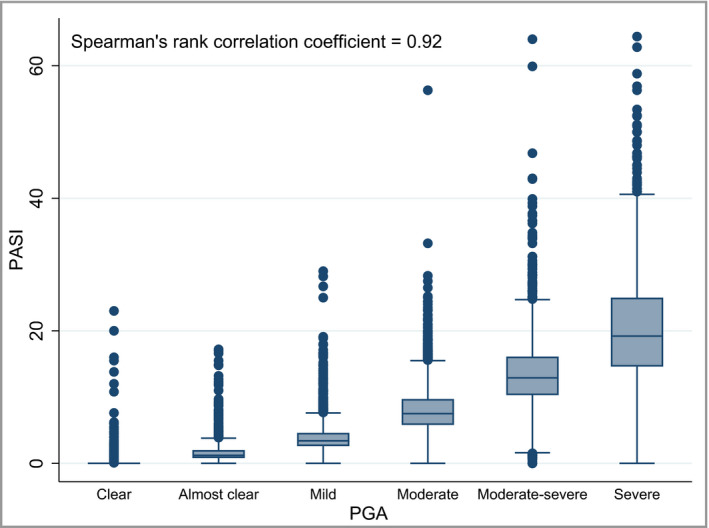
Correlation between Physician's Global Assessment (PGA) and Psoriasis Area and Severity Index (PASI). These data are derived from 23 475 occasions in 11 501 patients in which PASI and PGA were both recorded on the same day.

PASI and PGA were both recorded on the same day on 23 475 occasions in 11 501 patients. There is a strong positive Spearman's rank correlation coefficient of 0·92 between PASI and PGA (Fig. 2), as the median PASI decreases progressively from 19·2 (interquartile range 14·6–25·0) for PGA severe to 0 (interquartile range 0–0) for PGA clear (Table S2; see Supporting Information). However, there is large variability in the PASI score within each PGA category, as the range of PASI values overlaps across PGA categories (Fig. 2).

### Physician's Global Assessment clear or almost clear can be used interchangeably with Psoriasis Area and Severity Index ≤ 2

**Figure 3:**
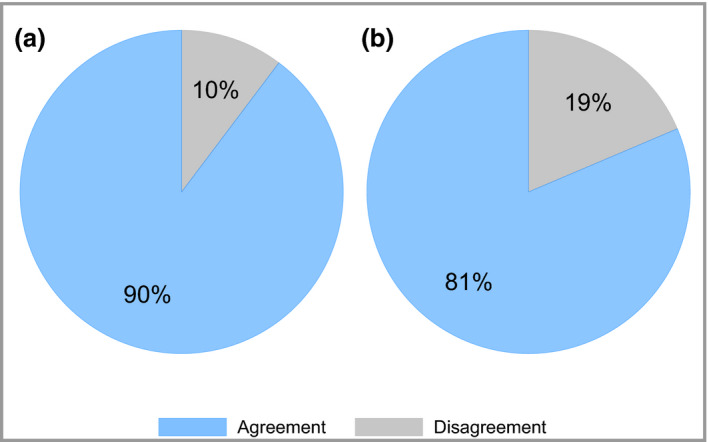
Agreement between (a) Physician's Global Assessment (PGA) clear or almost clear and Psoriasis Area and Severity Index (PASI) ≤ 2, and (b) PGA moderate–severe or severe and PASI ≥ 10. The blue segment represents the agreement between the two definitions and the grey segment represents the disagreement. The data in (a) are derived from 23 475 occasions in 11 501 patients in which PASI and PGA were both recorded on the same day. The data in (b) are derived from 10 154 patients for whom PASI and PGA were both recorded at baseline.

PASI ≤ 2 is consistent with PGA clear or almost clear in 90% of cases, with a Cohen's kappa of 0·79 (95% CI 0·78–0·80), indicating substantial agreement (Table 3, Fig. 3a). PASI ≤ 4 is concordant with PGA mild or better (i.e. PGA mild, almost clear or clear) in 90% of cases, with a Cohen's kappa of 0·77 (95% CI 0·77–0·78), indicating substantial agreement (Table 3).

**Table 3:** Comparison of Physician's Global Assessment (PGA) definitions of response with proposed absolute Psoriasis Area and Severity Index (PASI) definitions of response. These data are derived from 23 475 occasions in which PASI and PGA were both recorded on the same day, in 11 501 patients

		PASI ≤ 2	
		No	Yes
PGA clear or almost clear^a^	No	12 084 (51)	824 (4)
	Yes	1572 (7)	8995 (38)
		PASI ≤ 4	
		No	Yes
PGA clear, almost clear or mild^b^	No	7175 (31)	407 (2)
	Yes	2055 (9)	13 838 (59)

The data are presented as *n* (%). ^a^Agreement 90%, Cohen's kappa 0·79 (95% confidence interval 0·78–0·80). ^b^Agreement 90%, Cohen's kappa 0·77 (95% confidence interval 0·77–0·78).

### Physician's Global Assessment moderate–severe or severe is equivalent to the National Institute for Health and Care Excellence biologics eligibility criterion of Psoriasis Area and Severity Index ≥ 10

PASI and PGA were both recorded at baseline in 10 154 patients. In 81% of cases PGA moderate–severe or severe was consistent with the PASI‐based NICE eligibility criteria for biologic therapy (PASI ≥ 10) (Table 4, Fig. 3b). The Cohen's kappa of 0·46 (95% CI 0·44–0·48) indicates moderate agreement. In 3% of cases, eligibility would be gained under this PGA‐based definition, despite not satisfying current PASI‐based criteria. Conversely, 15% of people with PASI ≥ 10 would become ineligible for biologics according to this PGA definition.

**Table 4:** Comparison of the Psoriasis Area and Severity Index (PASI)‐based National Institute for Health and Care Excellence (NICE) biologics eligibility criterion (PASI ≥ 10) with the Physician's Global Assessment (PGA)‐based eligibility criterion. These data are derived from 10 154 patients in whom PASI and PGA were both recorded at baseline

		PASI ≥ 10, *n* (%)	
		No	Yes
PGA moderate–severe or severe	No	1255 (12)	1557 (15)
	Yes	338 (3)	7004 (69)

Agreement 81%, Cohen's kappa 0·46 (95% confidence interval 0·44–0·48).

### Sensitivity analysis

Sensitivity analyses show that all of the results are robust to the type of treatment, timing of assessments and baseline PASI (Table S3; see Supporting Information).

## Discussion

This study is the first real‐world systematic evaluation of absolute measures of disease control in psoriasis to date and is thus relevant for routine clinical practice, trials investigators and regulatory agencies. Using a multicentre cohort of more than 13 000 patients, we demonstrate that an absolute PASI ≤ 2 corresponds with PASI 90 response and is a relevant disease end point for treat‐to‐target approaches in psoriasis, obviating the need for baseline disease severity measurements. We also show that PASI and PGA are strongly correlated, and propose PGA moderate–severe/severe as an alternative eligibility criterion to PASI‐based definitions for biologics, and PGA clear/almost clear as an appropriate treatment end point.

Our study serves as the first validation of growing expert opinion supporting the use of absolute rather than relative measures of disease severity as treatment end points in psoriasis.^10^, ^19^ A recent consensus opinion paper based on the Delphi methodology proposed that absolute PASI ≤ 2 should be the pursued PASI goal, as it was felt to correlate better than relative PASI with the health‐related quality‐of‐life measure DLQI.^20^ Our data support current trends in trial practices, whereby the proportions of patients achieving absolute PASI values are increasingly reported as secondary end points due to the recognition that these values may be more clinically meaningful than relative PASI measures.^21^, ^22^

In daily practice, where baseline PASI is often lower than in clinical trials due to switching between systemic agents, achieving a PASI 90 response has been shown to represent an unrealistic treatment goal. A recent multicentre prospective study using the BioCAPTURE Dutch cohort showed that an absolute PASI ≤ 2 was more often achieved than PASI 90 at week 24 of biologic therapy (24·2% vs. 14·8%, respectively).^23^ This real‐world relative PASI response rate is substantially lower than those reported in randomized controlled trials of the same biologics (adalimumab, etanercept, infliximab, ustekinumab), in which PASI 90 was achieved in 20–58% of patients at weeks 16–28.^24^, ^25^, ^26^, ^27^ This underscores the relevance of absolute disease scores for defining clinically viable treat‐to‐target strategies (as derived in our study), and holds great promise for improving real‐world patient outcomes.

As the limitations of the PASI are well recognized,^28^, ^29^ the European Medicines Agency and U.S. Food and Drug Administration recommend that it is used in conjunction with PGA to assess efficacy and inform licensing of new treatments.^13^, ^30^ Clinicians are facing increasing time and resource pressures in the context of a rising global prevalence of psoriasis,^1^, ^31^ so PGA is often used in preference to PASI measurements in real‐world settings. We validate this approach by demonstrating a close correlation between PGA and PASI, irrespectively of treatment modality. This substantiates findings from a meta‐analysis of 30 randomized controlled trials of biologic agents in moderate–severe psoriasis (using varying PGA Likert scales), which demonstrated a correlation coefficient of 0·92 (*P* < 0·01) between PASI 75 responses and PGA clear or almost clear for study weeks 8–16.^29^ Assessment of PASI 90 responses was not within the scope of the meta‐analysis; however, PASI 90 has been subsequently shown to correlate significantly with an Investigator's Global Assessment (six‐point Likert scale) of clear or almost clear in a phase IIb trial of secukinumab 150 mg monthly dosing (48·1% clear or almost clear and 51·9% PASI 90 at week 12).^32^ Given that PGA clear or almost clear correctly classifies nearly all PASI 90 responses in our real‐world dataset, the use of PGA clear or nearly clear may be a justified descriptor of PASI 90 in routine practice.

Our proposed eligibility criterion for biologic therapy of PGA moderate–severe or severe is in keeping with U.S. guidelines^12^ and could be rapidly adopted into daily clinical practice due to the ease of measurement of categorical as opposed to quantitative variables. We have used the U.K. healthcare model to explore the potential impact of switching to the PGA. Despite not accounting for the extent of body surface involvement, our PGA‐based eligibility criterion would not result in a substantial increase in the number of eligible patients compared with the PASI‐based criterion used in current U.K. guidelines,^9^ and therefore would not be expected to have a major impact on current cost‐effectiveness modelling for biologic therapies. However, as 15% of eligible cases become ineligible for a biologic using this PGA criterion, we propose that at least one of PGA moderate–severe/severe and PASI ≥ 10 is considered the eligibility criterion in routine practice. Importantly, according to our proposed PGA criterion, individuals may be considered for biologic therapy if they have a lower PASI but severe, localized disease on sites that are associated with high functional impairment or distress (e.g. the face or genitals).^33^

The major strengths of this study are the large sample size, high external validity conferred by the participation of multiple centres across the U.K. and Republic of Ireland^14^ and fully independent data analysis. Detailed data capture occurred at any point in the treatment cycle, spanned a wide time frame (2005–2018) and allowed for analysis of both nonbiologic systemic and biologic therapies, which maximizes the generalizability of our findings.

The limitations of the study include the predominant inclusion of patients with moderate–severe disease, as the dataset relates to individuals qualifying for or already receiving a systemic agent. Correlation with patient‐reported measures such as DLQI was out of the scope of this study. Data were collected for other purposes, such as to justify commencing a biologic agent, which may introduce potential distortions in the dataset such as inflated baseline severity scores. The generalizability of our PGA data is limited by the lack of universal adoption of a single PGA score in routine practice and clinical trials, or by regulatory agencies.^34^ However, most do employ the six‐point score to rate disease severity from ‘clear’ to ‘severe’ that was used for our study.^13^, ^30^

As it is likely that PGA and PASI are simultaneously measured by the same assessor, the concordance between these scores may be inflated, as the first measurement may bias the second. The order in which the PASI and PGA are measured may also therefore be relevant. However, the potential influence of these factors on our results is limited by the consecutive measurement of scores throughout the treatment cycle for each individual.

Finally, our dataset is based on the currently available systemic and biologic therapies for psoriasis; however, the therapeutic armamentarium for psoriasis is undergoing a rapid expansion, with several agents recently approved for use or awaiting imminent approval.^3^ As these newer agents offer comparable or better efficacy rates compared with the analysed treatments, our proposed stringent end points are likely to remain relevant. We also demonstrated the robustness of our findings in different time windows, thereby indicating negligible influence of drug class.

In conclusion, as skin clearance or near clearance is now a realistic outcome for treatment of psoriasis, irrespectively of baseline disease severity, this study proposes up‐to‐date treatment goals based on real‐world data. We demonstrate that an absolute PASI ≤ 2 is a relevant and practical disease end point in psoriasis, which could be used to define treat‐to‐target approaches in routine clinical settings. If adopted, this paradigm shift in psoriasis care would align its clinical practice with that of other chronic diseases such as diabetes and hypertension, wherein patients are treated to the goal of ‘normalization’ in order to prevent end organ damage (e.g. cardiac events).^35^ Our data also highlight PGA severity scores as alternatives to PASI for treatment eligibility criteria and measures of response, which is likely to be highly clinically viable. Future analysis of patient and clinician acceptability of our proposed criteria will yield additional important data on clinical utility.

## Supporting information


**Table S1** Baseline characteristics of the BADBIR cohort: comorbidities and use of biologic and non‐biologic systemic therapies.
**Table S2** Descriptive statistics of Psoriasis Area and Severity Index by each Physician's Global Assessment score.
**Table S3** Sensitivity analyses.
