# Supplementary material for: Psoriasis treat to target: defining outcomes in psoriasis using data from a real‐world, population‐based cohort study (the British Association of Dermatologists Biologics and Immunomodulators Register, BADBIR)
Source: Br J Dermatol. 2019 Sep 10;182(5):1158–66. doi: 10.1111/bjd.18333 (PMC7317460; doi:10.1111/bjd.18333)
Supplement: Supplementary file 1 — Table S1 Baseline characteristics of the BADBIR cohort: comorbidities and use of biologic and non‐biologic systemic therapies. Table S2 Descriptive statistics of Psoriasis Area and Severity Index by each Physician's Global Assessment score. Table S3 Sensitivity analyses. [file BJD-182-1158-s001.doc]

**Supplementary Table 1: Baseline characteristics of the BADBIR cohort.**

|  | **Biologic cohort** | **Non-biologic systemic cohort** | **Overall** |
| --- | --- | --- | --- |
|  | (n=9201) | (n=4221) | (n=13422*) |
| **Biologic and non-biologic systemic therapies used at enrolment** |  |  |  |
| Acitretin | 0 (0.0%) | 773 (18.3%) | 773 (5.8%) |
| Adalimumab (Humira)  Apremilast | 4778 (51.9%)  0 (0.0%) | 0 (0.0%)  1 (<1%) | 4778 (35.6%)  1 (<1%) |
| Certolizumab | 2 (<1%) | 0 (0.0%) | 2 (<1%) |
| Ciclosporin | 0 (0.0%) | 1027 (24.3%) | 1027 (7.7%) |
| Clinical trial biologic / biosimilar | 4 (<1%) | 0 (0.0%) | 4 (<1%) |
| Efalizumab (Raptiva) | 25 (0.3%) | 0 (0.0%) | 25 (0.2%) |
| Etanercept (Enbrel) | 1531 (16.6%) | 0 (0.0%) | 1531 (11.4%) |
| Fumaric acid esters  Hydroxycarbamide  Infliximab (Remicade, Inflectra) | 0 (0.0%)  0 (0.0%)  213 (2.3%) | 303 (7.2%)  32 (0.8%)  0 (0.0%) | 303 (2.3%)  32 (0.2%)  213 (1.6%) |
| Methotrexate | 0 (0.0%) | 1999 (47.4%) | 1999 (14.9%) |
| PUVA | 0 (0.0%) | 86 (2.0%) | 86 (0.6%) |
| Secukinumab (Cosentyx) | 381 (4.1%) | 0 (0.0%) | 381 (2.8%) |
| Ustekinumab (Stelara) | 2267 (24.6%) | 0 (0.0%) | 2267 (16.9%) |
|  |  |  |  |
| **Number of co-morbidities** |  |  |  |
| 0 co-morbidities | 2849 (31.0%) | 1655 (39.2%) | 4504 (33.6%) |
| 1-2 co-morbidities | 5031 (54.7%) | 2106 (49.9%) | 7137 (53.2%) |
| 3-4 co-morbidities | 1174 (12.8%) | 417 (9.9%) | 1591 (11.9%) |
| > 5 co-morbidities | 147 (1.6%) | 43 (1.0%) | 190 (1.4%) |
|  |  |  |  |

* 1,371 patients switched from a non-biologic systemic to a biologic agent so are included in these summaries twice. There are therefore 12,051 unique patients overall.

**Supplementary Table 2: Descriptive statistics of PASI by each PGA score.** These data are derived from 23,475 occasions in 11,501 patients in which PASI and PGA are both recorded on the same day.

|  | **PASI** | |
| --- | --- | --- |
| **PGA** | **Mean (SD)** | **Median (IQR)** |
| **Severe**  (n=1233) | 20.5 (8.8) | 19.2 (14.6, 25.0) |
| **Moderate – Severe**  (n=2281) | 13.4 (5.6) | 12.9 (10.3, 16.1) |
| **Moderate**  (n=4068) | 8.0 (3.4) | 7.5 (5.8, 9.7) |
| **Mild**  (n=5326) | 3.8 (2.0) | 3.4 (2.6, 4.6) |
| **Almost Clear**  (n=6193) | 1.6 (1.3) | 1.2 (0.8, 2.0) |
| **Clear**  (n=4374) | 0.1 (0.7) | 0.0 (0.0, 0.0) |

**Supplementary Table 3: Sensitivity analyses.**

|  |  | **Percentage agreement**  **Cohen’s kappa (95% CI)**  **Number of observations** | | | |
| --- | --- | --- | --- | --- | --- |
| **Category** | **Sub-group** | **PASI 90**  **and**  **PASI ≤ 2** | **PASI 75**  **and**  **PASI ≤ 4** | **PGA Clear/ Almost Clear and**  **PASI < 2** | **PGA Moderate-Severe/Severe and PASI > 10** |
| **Treatment type** | Biologic cohort | 89%  0.78 (0.77,0.79)  16612 | 89%  0.76 (0.75,0.77)  16612 | 89%  0.78 (0.78,0.79)  16983 | 83%  0.46 (0.44,0.49)  6576 |
|  | Non-biologic systemic cohort | 92%  0.74 (0.72,0.76)  6889 | 86%  0.71 (0.70,0.73)  6889 | 91%  0.78 (0.76,0.79)  6492 | 79%  0.46 (0.43,0.49)  3578 |
| **Timing of assessment following start of treatment** | 6 months | 90%  0.78 (0.77,0.80)  7298 | 89%  0.78 (0.76,0.79)  7298 | 89%  0.78 (0.77,0.80)  7230 | NA |
|  | 12 months | 90%  0.79 (0.77,0.80)  7515 | 88%  0.75 (0.74,0.77)  7515 | 90%  0.80 (0.78,0.81)  7483 | NA |
| **Baseline PASI** | <10 | 79%  0.56 (0.53,0.58)  3905 | 69%  0.44 (0.42,0.46)  3905 | 89%  0.78 (0.76,0.80)  3508 | NA |
|  | 10-20 | 91%  0.80 (0.79,0.81)  14825 | 93%  0.85 (0.85,0.86)  14825 | 90%  0.80 (0.79,0.81)  13112 | NA |
|  | >20 | 95%  0.90 (0.88,0.91)  4771 | 89%  0.78 (0.77,0.80)  4771 | 91%  0.80 (0.78,0.82)  4172 | NA |
| **Time period of assessment (years)** | 2013- 2015 | 90%  0.78 (0.77,0.79)  11216 | 89%  0.77 (0.76,0.78)  11216 | 90%  0.80 (0.79,0.81)  10856 | 83%  0.50 (0.47,0.53)  4291 |
|  | 2016- 2018 | 89%  0.78 (0.76,0.80)  5481 | 88%  0.74 (0.72,0.76)  5481 | 91%  0.81 (0.80,0.83)  5413 | 84%  0.57 (0.52,0.62)  1458 |
| **Timing of baseline PASI assessment** | On treatment start date | 90%  0.76 (0.75,0.78)  5989 | 87%  0.74 (0.72,0.76)  5989 | 90%  0.79 (0.77,0.80)  5328 | 79%  0.48 (0.45,0.51)  2889 |
|  | Prior to treatment start date | 90%  0.78 (0.77,0.79)  17512 | 89%  0.76 (0.76,0.77)  17512 | 90%  0.79 (0.78,0.80)  18147 | 82%  0.45 (0.42,0.47)  7265 |

NA = not applicable as based only on baseline scores
